# Direct T-2 Toxicity on Human Skin—Fibroblast Hs68 Cell Line—In Vitro Study

**DOI:** 10.3390/ijms23094929

**Published:** 2022-04-29

**Authors:** Edyta Janik-Karpinska, Michal Ceremuga, Magdalena Wieckowska, Monika Szyposzynska, Marcin Niemcewicz, Ewelina Synowiec, Tomasz Sliwinski, Michal Bijak

**Affiliations:** 1 Biohazard Prevention Centre, Faculty of Biology and Environmental Protection, University of Lodz, Pomorska 141/143, 90-236 Lodz, Poland; edyta.janik.karpinska@edu.uni.lodz.pl (E.J.-K.); magdalena.wieckowska@edu.uni.lodz.pl (M.W.); marcin.niemcewicz@biol.uni.lodz.pl (M.N.); 2 Military Institute of Armament Technology, Prymasa Stefana Wyszyńskiego 7, 05-220 Zielonka, Poland; ceremugam@witu.mil.pl; 3 CBRN Reconnaissance and Decontamination Department, Military Institute of Chemistry and Radiometry, Antoniego Chrusciela “Montera” 105, 00-910 Warsaw, Poland; m.szyposzynska@wichir.waw.pl; 4 Laboratory of Medical Genetics, Faculty of Biology and Environmental Protection, University of Lodz, Pomorska 141/143, 90-236 Lodz, Poland; ewelina.synowiec@biol.uni.lodz.pl (E.S.); tomasz.sliwinski@biol.uni.lodz.pl (T.S.)

**Keywords:** T-2 toxin, skin, Hs68 cell line, cytotoxicity, necrosis

## Abstract

T-2 toxin is produced by different *Fusarium* species, and it can infect crops such as wheat, barley, and corn. It is known that the T-2 toxin induces various forms of toxicity such as hepatotoxicity, nephrotoxicity, immunotoxicity, and neurotoxicity. In addition, T-2 toxin possesses a strong dermal irritation effect and can be absorbed even through intact skin. As a dermal irritant agent, it is estimated to be 400 times more toxic than sulfur mustard. Toxic effects can include redness, blistering, and necrosis, but the molecular mechanism of these effects still remains unknown. This in vitro study focused on the direct toxicity of T-2 toxin on human skin—fibroblast Hs68 cell line. As a result, the level of toxicity of T-2 toxin and its cytotoxic mechanism of action was determined. In cytotoxicity assays, the dose and time-dependent cytotoxic effect of T-2 on a cell line was observed. Bioluminometry results showed that relative levels of ATP in treated cells were decreased. Further analysis of the toxin’s impact on the induction of apoptosis and necrosis processes showed the significant predominance of PI-stained cells, lack of caspase 3/7 activity, and increased concentration of released Human Cytokeratin 18 in treated cells, which indicates the necrosis process. In conclusion, the results of an in vitro human skin fibroblast model revealed for the first time that the T-2 toxin induces necrosis as a toxicity effect. These results provide new insight into the toxic T-2 mechanism on the skin.

## 1. Introduction

**Figure 1:**
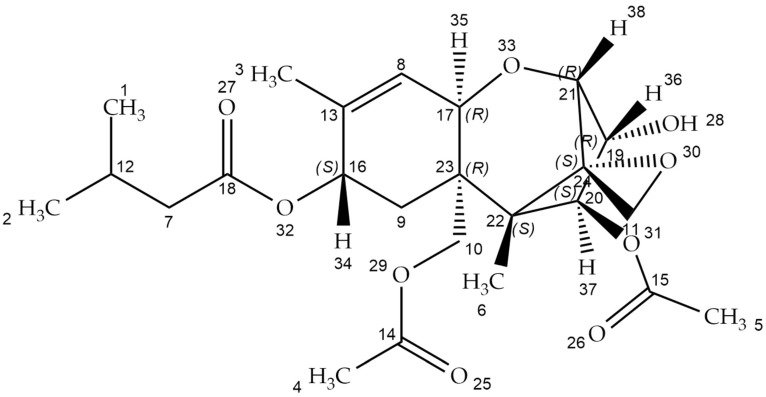
T-2 toxin chemical structure (structure generated from InChI code. Available online: https://pubchem.ncbi.nlm.nih.gov/ (accessed on 21 April 2022)).

T-2 toxin (Figure 1)—((1R,9R,10R,11S,12R)-11-acetyloxy-2-(acetyloxymethyl)-10-hydroxy-1,5-dimethylspiro(8-oxatricyclo(7.2.1.02,7])dodec-5-ene-12,2’-oxirane)-4-yl)-3-methylbutanoate (according the IUPAC nomenclature)—is the most toxic member of the fungal secondary metabolite belonging to the type A trichothecenes [1]. Trichothecenes are tetracyclic sesquiterpene compounds that consist of a trichothecene core with epoxy rings at the C-12 and -13 positions. This ring is responsible for toxicological activity. Trichothecenes are classified based on the carbonyl group at the 8-position, macrolide rings at the 4- and 5-positions, and the number of epoxy rings. Trichothecene-producing genera include *Fusarium*, *Myrothecium*, *Spicellum*, *Stachybotrys*, *Cephalosporium*, *Trichoderma*, and *Trichothecium* [2,3].

The T-2 toxin is produced mainly by *Fusarium* species (*F. poae*, *F. sporotrichioides*, *F. tricinctum*) and is a major crop and fodder pollutant. It can infect corn, barley, and wheat, both in the field and in wet storage conditions. Consumption of mycotoxin-contaminated cereal-based food and feed is a potential hazard for human and animal health [4]. The conditions which are optimal for enhancing toxin production are: substrate humidity (10–20%), relative humidity (≥70%), temperature (0 to 50 °C, depending on the fungus species), and oxygen availability [5].

The T-2 toxin has a low molecular weight of about 466.51 Da. It is also nonvolatile, insoluble in water, and highly resistant to degradation in different environmental conditions such as heat and UV light. It generates problems with toxin deactivation, however, the decontamination process is effective in strong acid or alkaline conditions [6]. The chemical structure of T-2 is characterized by the hydroxyl (OH) group at the C-3 position, acetyloxy (-OCOCH_3_) groups at the C-4 and C-15 positions, an atom of hydrogen at the C-7 position, and an ester-linked isovaleryl (OCOCH_2_CH(CH_3_)_2_) group at the C-8 position. The presence of hydroxyl groups, the structure, and the side-chain position affect the T-2 toxin’s biological activity [7,8].

The T-2 toxin is readily absorbed by various modes, including topical, oral, and inhalational routes. Unlike most typical biotoxins that do not affect the skin, T-2 is a potent, strong skin irritant and can be absorbed through intact skin causing systemic toxicity. As a blistering and dermal irritant agent, it is supposed to be 400 times more intoxicating than sulfur mustard (mustard gas, yperite), which is a chemical warfare agent [7]. In addition, the toxicity of the T-2 toxin by inhalation route is similar to that observed in mustards or lewisite. Therefore, the T-2 toxin’s properties are more similar to chemical agents than biological toxins. Dermal irritating properties of the T-2 toxin have been studied in different experimental animal models, e.g., rats [9], rabbits [10], guinea pigs [11], and cynomolgus monkeys [12]. In the case of poultry, dermatoxic effects are characterized as fatal ulceration or necrohemorrhagic dermatitis. Some animals exhibit comb cyanosis and depigmentation of the leg skin [13]. Agrawal et al. showed that in the mice model, T-2-mycotoxin-induced skin inflammation and cutaneous injuries occur [14].

In the late 1940s, scientists in the Soviet Union coined the term stachybotryotoxicosis to characterize an acute syndrome involving pharyngitis, bloody rhinorrhea, dyspnea, tussis, and fever resulting from mycotoxin inhalation. Alimentary toxic aleukia (ATA), a disease responsible for the losses of thousands of Soviet Union civilians during World War II, was caused by consumption of wheat that was unintentionally contaminated with *Fusarium* fungi. The victims developed a protracted lethal illness with a disease pattern similar to ATA [15].

Based on extensive eyewitness and victim accounts, it had been established that the T-2 toxin was used during the military conflicts in Laos, Cambodia, and Afghanistan from 1975 to 1981. The aerosolized form of the T-2 toxin was delivered by low-flying aircraft in the form of yellow oily droplets. Witnesses called the event “yellow rain” due to sticky, yellow drops of liquid that sounded similar to rain as they fell to the ground. It is estimated that exposure to “yellow rain” caused more than 6300 death in Laos, 3000 in Afghanistan, and 1000 in Cambodia [16,17]. The first symptoms appeared after a few minutes to an hour and included skin burning pain, tenderness, redness, and blistering. In fatal cases, progression of skin necrosis with leathery blackening and sloughing of large areas of skin occurred. Nasal contact was manifested by sneezing, pain, epistaxis, and rhinorrhea. Nausea, vomiting, diarrhea, and abdominal pain occurred in gastrointestinal toxicity. Systemic toxicity was manifested by weakness, ataxia, and loss of coordination. Additionally, in lethal cases, hypothermia, tachycardia, and hypotension were reported. Death occurred after a few minutes, hours, or days [18].

For this reason, it was decided to perform a study aimed at the direct toxicity action of the T-2 toxin on a cellular model of human skin—fibroblast cell line Hs68. The level of toxicity of the T-2 toxin as well as its cytotoxic mechanism of action were determined.

## 2. Results

### 2.1. Cell Viability

The cytotoxic effect of the T-2 toxin was evaluated in a normal human fibroblast cell line (Hs68) by two independent methods (trypan blue and MTT) which are based on different biochemical mechanisms. During the cytotoxicity assays for both methods, the dose-dependent cytotoxic effect of the T-2 toxin in the tested cell line was observed. Additionally, the cytotoxic effect was higher after 48 h of exposure in comparison to 24 h of time. The results in the form of cytotoxicity curves are presented in Figure 1.

**Figure 2:**
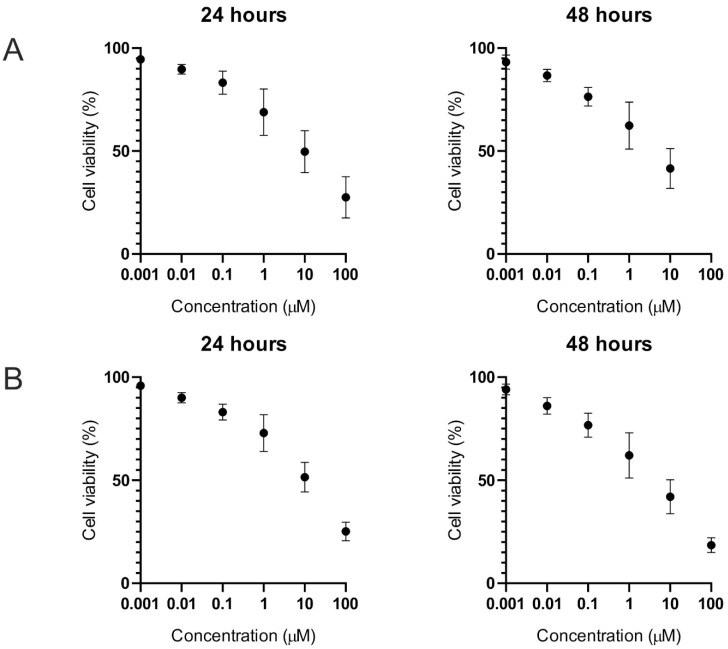
The T-2 toxin’s effect (in a concentration range from 0.001 to 100 µM) on Hs68 cell viability. Cell viability was estimated by using trypan blue (**A**) and MTT (**B**) methods. The data represent cell viability curves obtained from six independent measurements (*n* = 6).

Using an online tool (https://www.aatbio.com/tools/ec50-calculator (accessed on 21 April 2022))—Quest Graph™ EC50 Calculator—AAT Bioquest (Sunnyvale, CA, USA)—the T-2 toxin effective concentration 50 (EC50) parameters for the Hs68 cell line were obtained. The calculated values for the trypan blue method (Figure 2A) were 22.71 µM for 24 h of treatment and 7.81 µM for 48 h of treatment. The calculated results for the MTT test (Figure 2B) were 25.98 µM for 24 h of treatment and 10.35 µM for 48 h of treatment.

### 2.2. Cellular ATP Level

**Figure 3:**
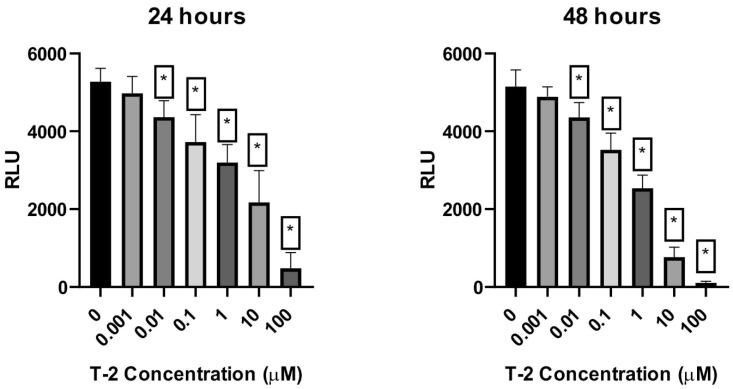
The T-2 toxin effect on the necrosis of ATP-level Hs68 cells estimated by the bioluminometry method. Values: means ± SD (*n* = 6). * *p* < 0.001. 0: untreated (control) cells; 0.001: concentration of toxin—0.001 µM; 0.01: concentration of toxin—0.01 µM; 0.1: concentration of toxin—0.1 µM; 1: concentration of toxin—1 µM; 10: concentration of toxin—10 µM; 100: concentration of toxin—100 µM.

Measurement of the level of ATP is the most sensitive, reliable, and convenient method for monitoring active cell metabolism. Using the bioluminometry method, the relative level of ATP in Hs68 cell suspension was determined. The treatment of the cell line resulted in a dose-dependent and time-dependent decrease in the level of luminescence, which directly corresponded to the ATP level in the sample (Figure 3).

### 2.3. Apoptosis and Necrosis

#### 2.3.1. Annexin V and Propidium Iodide Staining

**Figure 4:**
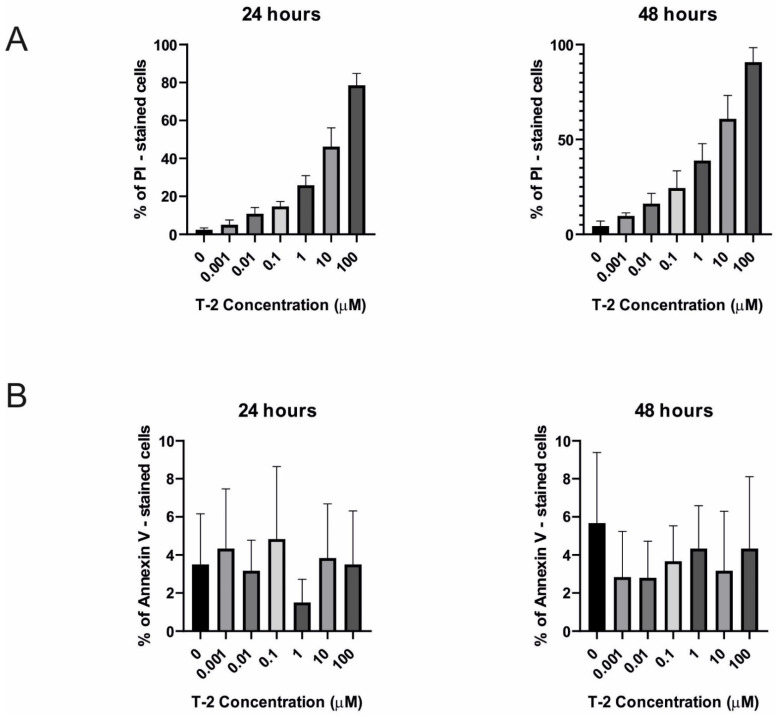
The T-2 effect on necrosis and apoptosis induction in Hs68 cells. The cell death pathway was assayed by flow cytometry with annexin V/propidium iodide staining after 24 h (**A**) and 48 h (**B**) of incubation with the toxin. Values: means ± SD (*n* = 6). 0: untreated (control) cells; 0.001: concentration of toxin—0.001 µM; 0.01: concentration of toxin—0.01 µM; 0.1: concentration of toxin—0.1 µM; 1: concentration of toxin—1 µM; 10: concentration of toxin—10 µM; 100: concentration of toxin—100 µM.

It was observed, using the double-staining flow cytometry method, that incubation of a normal human fibroblast cell line (Hs68) with T-2 mycotoxin resulted in both a dose-and time-dependent increase in propidium iodide (PI) fluorescence. It was related to the cells’ necrosis process. In Figure 3, the results obtained during this analysis are presented. In the highest tested concentration of toxin—100 µM—the % of PI-stained cells was 79% after 24 h of incubation and 93% after 48 h of incubation (Figure 4A). In all samples, any significant increase of annexin V fluorescence related to the apoptosis process in cells (Figure 4B) was not observed.

#### 2.3.2. Caspase-3/7 Pathway

**Figure 5:**
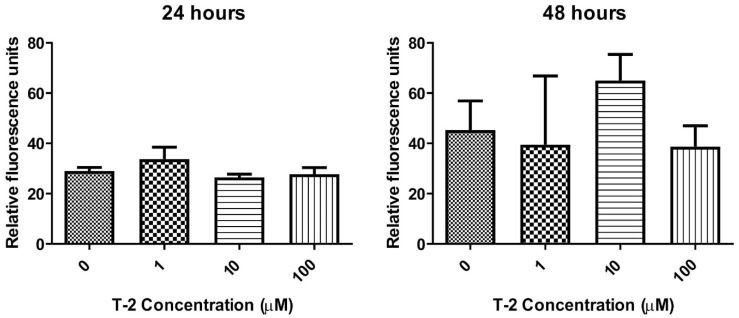
The T-2 effect on the caspase 3/7 pathway activation in Hs68 cells. Caspases’ proteolytic activity was measured by fluorescence of 485 nm/538 nm after 24 h and 48 h incubation with the toxin, respectively. Values means ± SD (*n* = 6). 0: untreated (control) cells; 1: concentration of toxin—1 µM; 10: concentration of toxin—10 µM; 100: concentration of toxin—100 µM.

In the next step of the study, the verification of the T-2 toxin’s ability to activate the caspase-3/7 pathway in human fibroblast cells was performed. During the fluorescence-based caspase activity estimation, it was observed that T-2 treatment of human fibroblast cells did not induce any changes in 590 nm fluorescence (Figure 5), which clearly indicated the absence of caspase 3/7 proteolytic activity in these cells.

#### 2.3.3. Cytokeratin 18 Concentration

**Figure 6:**
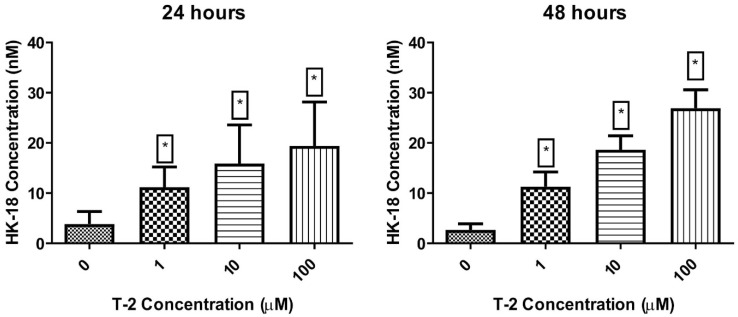
The T-2 effect on full-length Human Cytokeratin 18 concentration in Hs68 cells. Concentration was measured using ELISA method with absorbance measured at λ = 450 nm. Values: means ± SD (*n* = 6). * *p* < 0.001. 0: untreated (control) cells; 1: concentration of toxin—1 µM; 10: concentration of toxin—10 µM; 100: concentration of toxin—100 µM.

To confirm the activation of the necrotic pathway by the T-2 toxin in Hs68 cells, the analysis focused on examining full-length Human Cytokeratin 18 (CK18) concentration in cell culture supernatants was performed. The cell line treatment resulted in dose and time-dependent increased concentration of released Human CK18 (Figure 6). Additionally, in all tested concentrations of T-2 toxin, statistical significance (*p* < 0.001) of differences in comparison to the control sample was observed.

## 3. Discussion

Human skin consists of a few tissue layers: epidermis, dermis, and subcutaneous tissue. The first, the epidermis, is the outermost layer. It is composed of melanocytes responsible for pigment production, keratinocytes for protein secretion, and lipids forming the extracellular matrix (ECM), as well as Langerhans cells (LCs) involved the antigen (Ag) presentation. Below the epidermis is the dermis, consisting of connective tissue, which provides skin elasticity and tensile strength through the ECM. The skin’s innermost layer is the subcutaneous tissue composed of macrophages that eliminate pathogens, fibroblasts producing ECM proteins, and adipocytes [19].

The T-2 mycotoxin, in contrast to most biological toxins, is a strong skin irritant, and it may cause toxicity through contact with intact skin [20]. Associated symptoms of transdermal exposure are burning pain, redness, tenderness, and blisters, leading to skin necrosis. In addition to the above-presented symptoms, the affected skin area is affected by leathery blackening and sloughing off of exposed skin areas. The immediate toxic effect is characterized by the onset of symptoms seconds after exposure; however, lethal effects are only achieved with a high dose of toxin T-2 [7]. Moreover, T-2 toxic effects expressed by dystrophia in various organs (kidney, liver, heart, peripheral ganglia of the vegetative nervous system), digestive system ulceration and necrosis, hemorrhagic inflammation, diathesis, and injury of blood vessel walls have been observed [21].

Most of the information about T-2 blistering potential is based on observation, and there is no research data concerning in vitro studies aiming to establish the toxic effect of the T-2 toxin on human fibroblast cell line—Hs68.

The mechanism of the toxic effect of T-2 is based on the inhibition of protein synthesis, reduction of lymphocyte proliferation, immunosuppression by inhibiting the production of antibodies (Abs), and impaired development of dendritic cells (DCs) [22,23]. In vitro and animal models studies have suggested a pro-apoptotic effect of the toxin through oxidative damage of cellular components, mainly mitochondria and the rough endoplasmic reticulum. The T-2 toxin inhibited cellular energy by blocking the activity of metabolically important enzymes, leading to a reduction in protein synthesis in the mitochondria, and inhibiting the oxidation of malate, pyruvate, and succinate. It has been noted that certain antioxidants such as lycopene, vitamin E, and rutin counteracted the T-2 toxic effects by inhibiting lipid peroxidation, regulating glutathione metabolism, and enhancing antioxidant enzyme activity [7,24,25,26]. Thus, the cytotoxic activity of T-2 is associated with oxidative stress induction leading to damage of DNA and lipids, as well as protein synthesis inhibition [27].

For these reasons, this study focused on the evaluation of T-2 toxicity in in vitro conditions on human skin using an Hs68 human dermal fibroblast cell line. The evaluation of the cytotoxic effect demonstrated the high toxic properties of the T-2 toxin in two independent assays. The EC_50_ parameter is very often used as a compound toxicity indicator as well as in pharmacology, because it serves as an indication of drug potency. The calculated values of this parameter clearly demonstrate that T-2 as a dermal agent can be very effective.

ATP is an intracellular energy transfer molecule which has been found in all known forms of all living things. The properties of ATP are related to phosphate groups that link through phosphodiester bonds, which are associated with electronegative charges exerting a repelling force. The process of ATP hydrolysis to ADP is energetically favorable, yielding Gibbs-free energy of −7.3 cal/mol. In eukaryotic cells, the main place of ATP synthesis is the mitochondrial matrix, generating approximately thirty-two ATP molecules per one glucose molecule that is oxidized [28]. ATP is involved in a variety of enzymatic reactions to maintain normal life activities. When normal cells undergo apoptosis and necrosis, the content of ATP will be characteristically changed. Thus, ATP has been widely accepted as a valid marker of metabolically active cells [29]. One of the best measurement methods of intracellular ATP is using firefly luciferase, which takes part in the oxidation of D-luciferin to oxyluciferin [30]. In these experiments, it was confirmed that the T-2 decreases the level of intracellular ATP in a dose-dependent manner. In the probes with the maximum tested toxin concentration (100 µM), after 48 h, an almost total reduction in the ATP level was observed. These results confirmed cytotoxic analysis of the T-2 toxin’s potential to damage the human skin fibroblast cell line—Hs68.

Necrosis is uncontrolled cell death induced by external injury, such as inflammation, ischemia, hypoxia, hypoglycemia, toxin exposure, extreme temperature changes, nutrient deprivation, or ROS-induced injury [31]. The other cell death form, apoptosis, takes place as the physiological state of the organism during development and ageing and is a homeostatic mechanism to maintain cell populations in different tissues. During the apoptotic process, there is no inflammatory reaction. This is related to three main characteristics of apoptosis: (i) apoptotic cells do not release their cellular ingredients into the extracellular matrix; (ii) the cell fragments are rapidly phagocytosed by neighboring cells and, (iii) the engulfing cells do not produce anti-inflammatory cytokines [32]. In contrast to apoptosis, necrosis is a passive process requiring only minimal energy. It does not require new protein synthesis and is not regulated by any homeostatic mechanism [33]. This form of cell death is accompanied by extensive swelling of the cell (as it does not maintain homoeostasis with its environment), distension of different cellular organelles, clumping and random degradation of nuclear DNA, and extensive loss of the intracellular contents [34,35]. Necrosis is a consequence of extensive crosstalk among several biochemical and molecular events at different cellular levels. It often involves the upregulation of numerous pro-inflammatory proteins and compounds, such as nuclear factor-κB (NF-κB), resulting in a cascade of inflammation and tissue damage [36]. In addition, necrosis can be accompanied by ATP depletion [35]. Necrosis may be the result of the toxicity of other mycotoxins, as shown by the following studies. Dolensek et al. showed the effects of a diet contaminated with zearalenone (ZEN), deoxynivalenol (DON), and fusaric acid (FA) on gilts’ liver and their suckling piglets. The experimental diet contained 0.09 mg ZEN, 5.08 mg DON, and 21.6 mg FA per kg of feed. The gilts were fed the experimental diet for 54 ± 1 day. The results showed histopathological liver changes and a significant increase in hepatocellular necrosis [37]. In a different study, piglets receiving a diet multi-contaminated with DON (3 mg per kg of feed), ZEN (1.5 mg per kg of feed), and nivalenol (NIV) (1.3 mg per kg of feed) for 28 days exhibited focal liver necrosis [38]. Antonissen and colleagues conducted a study aimed at examining the effect of DON on necrotic enteritis development in broiler chickens. Results demonstrated that the intake of DON-contaminated feed at concentrations below the EU maximum guidance level of 5.000 µg/kg feed significantly increased the number of broiler chickens affected with necrotic enteritis. It lead to an altered intestinal barrier function and resulted in an increased permeability of the intestinal wall [39]. A study with male rats as an animal model demonstrated the neurodegenerative properties of aflatoxin B1 (AFB1). AFB1 was orally administered to male rats at a dose of 0.025 mg/kg of body weight for 90 days. The oral intake of AFB1 caused a time-dependent severity of histopathological impairments in the brain tissue, including spongiform necrosis [40]. A different study concerned the effects of fumonisin B1 (FB1) on the BV-2 cell line and primary murine astrocytes. Cells were exposed to various concentrations of FB1 for 4 to 8 days. Results showed that FB1 induces necrotic cell death in both BV-2 and primary astrocytes [41]. Kupski et al. studied the toxic effects of ochratoxin A (OTA) in human neutrophils in vitro. The study showed that OTA activates neutrophils culminating in cell death by necrosis. According to the results, OTA induces the release of Ca^2+^ from internal stores, leading to an increase in intracellular Ca^2+^ and human neutrophils’ oxidative burst followed by depletion of ATP levels and changes in mitochondrial potential, leading to cell death through necrosis [42].

The double-staining cytometry method was used to estimate which potential mechanism of cell death is induced by the T-2 toxin. An annexin V and PI staining kit has been specifically designed for the identification of apoptotic and necrotic cells. Annexin V is a member of the annexin family of intracellular proteins that binds to phosphatidylserine (PS) in a calcium-dependent manner. The surface expression of phosphatidylserine on the cellular membrane lipid is a characteristic event of the apoptotic process, which leads to the recognition of apoptotic cells for phagocytosis [43]. PI is a fluorescent dye that binds to DNA. Early apoptotic cells will exclude PI, while late-stage apoptotic cells and necrotic cells will stain positively due to the passage of these dyes into the nucleus, where they bind to DNA. The flow cytometry analysis clearly demonstrated the lack of presence of PS on cells treated by the T-2 toxin, simultaneously showing the binding of PI to the cells’ genetic material (Figure 4), which suggests induction of necrosis by this compound. To verify the absence of induction of the apoptosis, another method—evaluation of activation caspases 3 and 7—was used. These protease enzymes cleave proteins at positions containing aspartic acid residues. The specificity of different caspases is dependent on the recognition of neighboring amino acids. Furthermore, activation of caspases appears to lead to an irreversible induction of programmed cell death [32]. This analysis confirmed the purposes concerning no signs of an apoptotic pathway in T-2-induced Hs-68 cell death (Figure 5). The increased cell permeability without significant increases in caspase 3/7 activation indicated that apoptosis is not involved in the death of these cells.

For final verification of observations, the quantitative analysis of the presence of full-length human cytokeratin was performed. CK18 is a cytoskeletal protein and the main intermediate filament family member expressed in the liver. The full-length form is liberated from necrotic cells, whereas a caspase-cleaved fragment is a product of the structural changes that occur during apoptosis [44]. It was observed that supernatant concentration of CK18 in cell samples increased in a dose-dependent and time-dependent manner (Figure 5). This observation confirmed hypotheses based on other analyses presented in the current study that the T-2 toxin has very strong toxic potential against human skin fibroblast cells and is also a necrosis-induced factor. Other studies have shown that the T-2 toxin activated p38MAPK (SAPK2) and/or Jun N-terminal kinase 1 (JNK1), which induced MAP kinases, including ERK 1/2 [24]. Moreover, it has also been shown that the T-2 toxin stimulates the caspase-3-dependent apoptotic pathway through the upregulation of Fas and p53, which leads to an enhancement of the Bax/Bcl-xL and Bax/Bcl2 ratios [45]. In turn, in this work, it was noted that the effect of T-2 on Hs68 cells was independent of the activation of caspase 3/7.

## 4. Materials and Methods

### 4.1. Reagents

Dimethyl sulfoxide (DMSO) and T-2 Toxin from Fusarium sp. (cat. No T4887) were obtained from Sigma-Aldrich Chemical Co. (St. Louis, MO, USA). Penicillin–streptomycin mixture, Dulbecco’s Modified Eagle Medium (DMEM) with 4.5 g/L Glucose and with L-Glutamine, heat-inactivated fetal bovine serum (FBS), and PBS (1X) without calcium or magnesium were purchased from Lonza (Basel, Switzerland). A cell viability kit with trypan blue dye and cell counter slides was obtained from BIO-RAD (Hercules, CA, USA). A FITC Annexin V Apoptosis Detection Kit I was obtained from Becton Dickinson (Franklin Lakes, NJ, USA). The MTT (3-(4,5-Dimethylthiazol-2-yl)-2,5-Diphenyltetrazolium Bromide) and CellEvent™ Caspase-3/7 Green Flow Cytometry Assay Kit were obtained from Thermo Fisher Scientific (Waltham, MA, USA). All other chemicals were reagent-grade or the highest-quality available.

### 4.2. Cellular Material and Culturing Procedure

In this study, the human foreskin fibroblast line Hs68 (ATCC^®^ CRL-1635™) obtained from the American Type Culture Collection (ATCC™, Manassas, VA, USA) was used. Hs68 cells were cultured in DMEM medium supplemented with 100 units of potassium penicillin and 100  μg of streptomycin sulphate per 1  mL of culture media and 10% (*v*/*v*) FBS. The cell growing process was performed in a humidified incubator at 37 °C and 5% CO_2_. To perform the analysis, cells were seeded at 3 × 10^6^ cells per well and were left in the incubator for 12 h before treatment procedures. Next, the cell samples were incubated with T-2 toxin with a concentration range of 0.001 to 100 µM for 24 h and 48 h.

### 4.3. Cell Viability Determination

The viability of T-2-treated cell samples was evaluated via two different methods. The first method was the trypan blue dye exclusion test. Analysis of viability via this method was performed using the BIO-RAD TC20 automated cell counter (Hercules, CA, USA), according to the manufacturer’s protocol. Cell viability was expressed as a percentage relative to the untreated (control) cells, defined as 100%. The second test was based on measuring cell metabolic activity, which reduces the tetrazolium dye MTT to its insoluble formazan. During this assay, the MTT (0.5 mg/mL) was added to all cell samples and incubated for 4 h at 37 °C. Next, the MTT solution was discarded carefully, and the formed formazan crystals were dissolved in DMSO. The amount of formed formazan crystals was measured calorimetrically at a wavelength of 570 nm with background subtraction at 630 nm on Microplate Reader—BioTek Synergy HT (BioTek Instruments, Winooski, VT, USA). Cell viability was expressed as a percentage relative to the untreated (control) cells, defined as 100%. Half maximal effective concentration (EC_50_) parameters were calculated using “Quest Graph™ EC500 Calculator” (Bioquest Inc., San Francisco, CA, USA, https://www.aatbio.com/tools/ec50-calculator (accessed on 19 March 2022)).

### 4.4. Analysis of Cellular ATP Level Using the Bioluminometry Method

The ATP level in Hs68 cells was evaluated using the bioluminometry method. This method is based on the measurement of light emission that arises during enzymatic degradation of ATP. Luciferase (via oxidation of luciferin) decomposes ATP molecules into AMP. This reaction is accompanied by the emission of light photons with an electromagnetic wavelength of 562 nm. Analysis of cells’ metabolic activity via this method was performed using the Hygiena SystemSURE Plus™ (Camarillo, CA, USA). The 100 µL of cell suspension (3 × 10^6^ cells/mL) in sterile DMEM was inserted in the Hygiena AquaSnap™ Total tubes (Camarillo, CA, USA). After that, the tubes were activated by breaking a Hygiena Snap Valve™ (Camarillo, CA, USA) and bending the bulb forward and backward to expel all liquid reagent into the tube. The tube nest was shaken for 5 s to mix the sample. The measurements were performed according to the manufacturer’s guidelines. The results were expressed as relative light units (RLU). The baseline was calibrated using the pure DMEM solution.

### 4.5. Apoptosis/Necrosis Assay—Annexin V Binding and Propidium Iodide Staining

The presence of apoptotic cells was evaluated based on the exposition of phosphatidylserine residues by flow cytometry method using FITC Annexin V Apoptosis Detection Kit I. After the culturing procedure, the cells were washed twice in cold PBS, then suspended in 100  µL 1× annexin-binding buffer (cell density: 1 × 10^5^) and transferred to a standard cytometric tube. Next to the cell suspension, the mixture of 5 µL of FITC Annexin V and 5 µL of propidium iodide (PI) was added, and probes were incubated at 37 °C in an atmosphere of 5% CO_2_. After 15 min of incubation, 400  μL of the 1× annexin-binding buffer was added, and samples were analyzed by flow cytometry with the PARTEC CUBE 6 (Görlitz, Germany) flow cytometer using 488 nm excitation. Gates for PI (630 nm Longpass filter) and FITC (536/40 nm filter) fluorescence were estimated based on the fluorescence of unstained probes. The apoptotic index was calculated as the mean percentage of apoptotic cells in 5 × 10^4^ cells measured in each experiment. All data analysis was performed in CyFlow version 1.5.1.2—PARTEC (Görlitz, Germany).

### 4.6. Determination of Activity of Caspase-3/Caspase-7

Flow cytometric detection of activated caspase-3 and caspase-7 in apoptotic cells was performed using the CellEvent™ Caspase-3/-7 Green Flow Cytometry Assay Kit. For this analysis, cells treated with the T-2 toxin were washed twice in cold PBS and suspended (cell density: 1 × 10^5^) in 1 mL PBS in an Eppendorf tube. In the next step, to each tube, 1 µL of CellEvent™ Caspase-3/-7 Green Detection Reagent was added and mixed gently and left at 37 °C in an atmosphere of 5% CO_2_. After the incubation procedure (30 min), 1 µL of the 1 mM SYTOX™ AADvanced™ (prepared in DMSO) was added and left in the dark, RT for 5 min. The final step of the analysis was performed using 96-well black plates (Greiner Bio-One, Kremsmünster, Austria), where 50 µL of samples was transferred to each well. Fluorescence was measured using the Bio-Tek Synergy HT Microplate Reader (Bio-Tek Instruments, Winooski, VT, USA), with filter pairs of 530 nm/590 nm and 485 nm/538 nm.

### 4.7. Concentration of Cytokeratin 18 Analysis in Cell Culture Supernatants

Human Cytokeratin 18 (CK18) concentration in cell culture supernatants obtained from the Hs68 cell line during the culturing process (with T-2 toxin) was analyzed using a sandwich ELISA kit—Human Cytokeratin 18/KRT18 ELISA (RayBiotech, Peachtree Corners, GA, USA). The whole procedure was performed according to the manufacturer’s protocol. Detection of absorbance measured at λ = 450 nm was performed using the SPECTROStar Nano Microplate Reader (BMG Labtech, Ortenberg, Germany). The concentration of CK18 concentration in cell culture supernatants was determined based on a standard curve expressed as ng/mL.

### 4.8. Data Analysis

All obtained experimental values were elaborated using Microsoft Excel (Redmond, WA, USA) and expressed as means ± standard deviations (SDs). The statistical analysis was performed using StatsDirect statistical software V. 2.7.2.—StatsDirect Ltd (Cheshire, UK). In the first step, all results were analyzed according to the normality of the distribution by the Shapiro–Wilk test. Next, the results were analyzed according to equality of variance via Levene’s test. The significance of the differences among the values was analyzed using ANOVA: Tukey’s range test (for data with a normal distribution and equality of variance) or the Kruskal–Wallis test; *p* < 0.05 was accepted as statistically significant [46,47].

## 5. Conclusions

We have demonstrated for the first time using an in vitro human skin fibroblast model that the T-2 toxin is able to induce the necrosis process. This is a very important step for research concerning the prevention of damages induced by this toxin. Further analysis at the molecular level has been planned for the next research project, which will also focus on T-2 toxin toxicity.

## Data Availability

Not applicable.
